# Environmental DNA-based biodiversity profiling along the Houdong River in north-eastern Taiwan

**DOI:** 10.3897/BDJ.12.e116921

**Published:** 2024-04-23

**Authors:** Chieh-Ping Lin, Chung-Hsin Huang, Trevor Padgett, Mark Angelo C. Bucay, Cheng-Wei Chen, Zong-Yu Shen, Ling Chiu, Yung-Che Tseng, Jr-Kai Yu, John Wang, Min-Chen Wang, Daphne Z. Hoh

**Affiliations:** 1 Genome and Systems Biology Degree Program, Academia Sinica and National Taiwan University, Taipei, Taiwan Genome and Systems Biology Degree Program, Academia Sinica and National Taiwan University Taipei Taiwan; 2 Biodiversity Research Center, Academia Sinica, Taipei, Taiwan Biodiversity Research Center, Academia Sinica Taipei Taiwan; 3 Biodiversity Program, Taiwan International Graduate Program, Academia Sinica, Taipei, Taiwan Biodiversity Program, Taiwan International Graduate Program, Academia Sinica Taipei Taiwan; 4 International Graduate Degree Program for Biodiversity, Tunghai University, Taichung, Taiwan International Graduate Degree Program for Biodiversity, Tunghai University Taichung Taiwan; 5 Department of Life Science, National Taiwan Normal University, Taipei, Taiwan Department of Life Science, National Taiwan Normal University Taipei Taiwan; 6 Marine Research Station, Institute of Cellular and Organismic Biology, Academia Sinica, Yilan, Taiwan Marine Research Station, Institute of Cellular and Organismic Biology, Academia Sinica Yilan Taiwan; 7 Institute of Oceanography, National Taiwan University, Taipei, Taiwan Institute of Oceanography, National Taiwan University Taipei Taiwan; 8 Institute of Cellular and Organismic Biology, Academia Sinica, Taipei, Taiwan Institute of Cellular and Organismic Biology, Academia Sinica Taipei Taiwan; 9 Zoological Institute, Christian-Albrechts University of Kiel, Kiel, Germany Zoological Institute, Christian-Albrechts University of Kiel Kiel Germany; 10 Taiwan Biodiversity Information Facility, Biodiversity Research Centre, Academia Sinica, Taipei, Taiwan Taiwan Biodiversity Information Facility, Biodiversity Research Centre, Academia Sinica Taipei Taiwan

**Keywords:** river ecosystem, species occurrence, metabarcoding, cytochrome c oxidase I gene, eukaryota

## Abstract

**Background:**

This paper describes two datasets: species occurrences, which were determined by environmental DNA (eDNA) metabarcoding and their associated DNA sequences, originating from a research project which was carried out along the Houdong River (猴洞坑), Jiaoxi Township, Yilan, Taiwan. The Houdong River begins at an elevation of 860 m and flows for approximately 9 km before it empties into the Pacific Ocean. Meandering through mountains, hills, plains and alluvial valleys, this short river system is representative of the fluvial systems in Taiwan. The primary objective of this study was to determine eukaryotic species occurrences in the riverine ecosystem through the use of the eDNA analysis. The second goal was, based on the current dataset, to establish a metabarcoding eDNA data template that will be useful and replicable for all users, particularly the Taiwan community. The species occurrence data are accessible at the Global Biodiversity Information Facility (GBIF) portal and its associated DNA sequences have been deposited in the European Nucleotide Archive (ENA) at EMBL-EBI, respectively. A total of 12 water samples from the study yielded an average of 1.5 million reads. The subsequent species identification from the collected samples resulted in the classification of 432 Operational Taxonomic Units (OTUs) out of a total of 2,734. Furthermore, a total of 1,356 occurrences with taxon matches in GBIF were documented (excluding 4,941 *incertae sedis*, accessed 05-12-2023). These data will be of substantial importance for future species and habitat monitoring within the short river, such as assessment of biodiversity patterns across different elevations, zonations and time periods and its correlation to water quality, land uses and anthropogenic activities. Further, these datasets will be of importance for regional ecological studies, in particular the freshwater ecosystem and its status in the current global change scenarios.

**New information:**

The datasets are the first species diversity description of the Houdong River system using either eDNA or traditional monitoring processes.

## Introduction

Environmental DNA (eDNA) metabarcoding is an emerging tool that can provide an accurate and comprehensive representation of biotic communities (Beng and Corlett 2020, Carvalho et al. 2021, Doi et al. 2021) by identifying multiple lineages from a single environmental sample (Taberlet et al. 2012). While the earliest uses of eDNA specifically focused on RNA detection of microbial communities (Ogram et al. 1987), in the last decade, eDNA has grown in scope to include macrobial communities and, as a result, has become an important tool in biodiversity assessments, biomonitoring and identifying temporal shifts in community assemblages of both terrestrial and aquatic ecosystems (Bista et al. 2017, Jeunen et al. 2019, West et al. 2020, Pawlowski et al. 2020, Burian et al. 2021, Gregorič et al. 2022).

Ecosystems are replete with the genetic material of both resident and transient species. This genetic material includes organismal and extra-organismal DNA from microorganisms and macroorganisms in the form of faeces, shed skin or hair, carcasses and living bodies (Stewart 2019). Using genomic approaches to detect these ‘genetic footprints’ of organism life from environmental samples, for example, water, snow, soil, air or leaf swabs (Stewart 2019), provides an opportunity to increase the detection rate (Goldberg et al. 2016) and increase temporal resolution (Ogram et al. 1987, Pawlowski et al. 2020, Taberlet et al. 2012, Alexander et al. 2023Taberlet et al. 2012,) of biological studies. Notably, eDNA analyses surpass conventional methods in terms of precision and resolution (e.g. Nakagawa et al. (2018), Rourke et al. (2021)). Furthermore, they exhibit greater efficiency in the context of both cost and time when compared to conventional biological monitoring approaches. These factors are crucial in advancing long-term ecological research on a global scale (Evans et al. 2017, Jerde 2019, Larson et al. 2020, Bruel and White 2021).

The application of eDNA for species composition assessments is flexible depending on the organisms of interest and may function as a general or targeted instrument (e.g. Thomas et al. (2019), Beng and Corlett (2020), Burian et al. (2021), Carvalho et al. (2021), Alexander et al. (2023)). Targeted analyses aim to identify the presence or absence of a specific focal species (i.e. “is the targeted species here”), while general analyses aim to identify community composition (i.e. “what species are here”). For both aims, by relying on the genetic footprints and not direct observation, eDNA can be superior to traditional methods by allowing for higher resolution of community assemblage (e.g. Alexander et al. (2022)); the enhanced detection of rare, migratory, elusive and cryptic species (e.g. Barnes et al. (2014), Beng and Corlett (2020), Rourke et al. (2021), Shen et al. (2022)) and the detection of community shifts (e.g. DiBattista et al. (2020)) and small scale spatial assemblage changeovers (e.g. Jeunen et al. (2019)). Environmental sampling, followed by eDNA analyses, provides a powerful approach for identifying a more comprehensive community complexion, which is crucial for researchers.

An ecosystem examination utilising eDNA necessitates exchanging the information collected in addition to the noted benefits and significance. For instance, there is undeniable value in sharing eDNA data in accordance with the FAIR (Findable, Accessible, Interoperable and Reusable) principles, thereby enriching our understanding of global biodiversity. The efforts of international organisations committed to advancing and promoting instruments for the exchange of eDNA data are noticeable in the discussions that commenced at the TDWG conference (Suominen et al. 2023).

Hence, in this paper, we describe two datasets:

1. A species occurrence dataset derived from the eDNA analysis of surface water from the Houdong River in north-eastern Taiwan and
2. their associated DNA sequences.

We aimed to determine community composition and diversity changes along a 6.5 km stretch of the river as it passes from headwaters (primary subtropical forest) through residential areas, aquaculture farms and agricultural fields (e.g. rice farm), before reaching the estuary/river mouth. This is the first known freshwater eDNA dataset originating from a representative turbulent river in Taiwan and will be important as baseline data for further studies and environmental monitoring of this ecosystem. Given the inexperienced DNA open data attempt within the Taiwan community, the study collaborates with the Taiwan Biodiversity Information Facility (TaiBIF) to establish a data template for the eDNA open data workflow. TaiBIF is amongst the most active data hosting centres and nodes of the Global Biodiversity Information Facility (GBIF). We expect that, through this collaboration, we can promote the dissemination and use of eDNA and DNA metabarcoding datasets from the Taiwan community.

## Sampling methods

### Study extent

**Figure 1.**
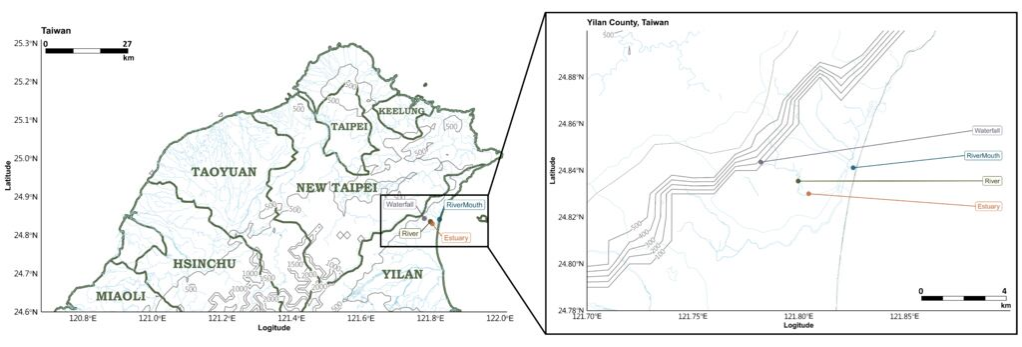
The four water sampling locations along the Houdong riverine system in Yilan County, Taiwan.

This was a one-time sampling event of water samples from four sites along the Houdong River in Jiaoxi Township, Yilan County, Taiwan (Fig. 1). The Houdong River is a popular tourist destination running across the Jiaoxi Township. The river system originates east of the Sidu Mountains and flows through primary and secondary forests, agricultural lands (rice), aquaculture farms and developed areas (light industrial and residential) until it eventually drains into the Pacific Ocean. Water samples were used for eDNA analysis and measurement of in-situ water quality.

### Sampling description

Water samples for eDNA were collected from near the surface of the river. Before the collection, the water containers were rinsed with the local water at each sampling site. Approximately 3 litres of water were collected for eDNA analysis. Some 200 ml of the collected water was used for water quality measurements. Water quality measurements were made using multiple hand-held probes on-site at each sampling site. Temperature, pH and dissolved oxygen were measured with a multi-parameter meter (Multiline® Multi 3620 IDS, WTW, Weilheim, Germany) equipped with an IDS pH electrode (SenTix 940, WTW) and an optical IDS dissolved oxygen sensor (FDO® 925, WTW). Turbidity was measured by a turbidity meter (TUB-430, EZDO, Taiwan). Salinity was determined by salinity refractometer (2491 MASTER-S/Milla Salinity Refractometer, Atago, Japan). Three replicate measurements of each parameter were taken. Water samples were transported to the Marine Research Station (MRS, Yilan County, Taiwan) of the Institute of Cellular and Organismic Biology for sample filtration. The water was first filtered through a 75 µm pore size sieve to eliminate larger particles. Afterwards, a 1 litre water sample from each site was filtered through a 0.22 µm filter and the sample kept on top of the filter membrane, under vacuum compression (PC651-0024, GeneDireX, USA). The filter membranes were placed in sterile Petri dishes and stored at -80°C until DNA extraction.

### Step description

**Wet lab process**

DNA was extracted at the Biodiversity Research Center (Academia Sinica, Taipei, Taiwan). Each filtered membrane was cut into quarters. Three of the four pieces of filtered membranes were used in the study as three experimental replicates. The final quarter was saved as the sample backup. DNA from each quarter membrane piece was extracted using the Presto™ Stool DNA Extraction Kit (STLD100, Geneaid Biotech Ltd., Taiwan) following the manufacturer's instructions (Instruction Manual Ver. 10.21.17). The quality and quantity of the extracted DNA was assessed using a Nanodrop 2000 (Thermo Fisher Scientific Inc., USA) and the Qubit 4 dsDNA High Sensitivity Assay Kit (Thermo Fisher Scientific Inc., USA).

The MinibarF1 (5'TCCACTAATCACAARGATATTGGTAC) and MinibarR1 (5'GAAAATCATAA TGAAGGCATGAGC) primers that were designed by Meusnier et al. (2008) were used to amplify the 5' region (ca. 120-150 bp) of the mitochondrial Cytochrome c oxidase I (COI) gene. The universality of the primers was recommended for distinguishing the highly diverse DNA from the environmental mixture. We conducted PCR using a one-step single-indexed approach, with a 13 bp tag attached to the MinibarR1 primer. The PCR reaction volume was 16 μl, which included 8 μl KAPA HiFi HotStart ReadyMix (KK2602, Roche Molecular Systems Inc., USA), 5 μl ddH20, 1 μl of each primer (10μM) and 1 μl of DNA template. To optimise the protocol, we performed a preliminary PCR using an annealing temperature gradient and found that 54°C gave the best results. The PCR mixture was denatured at 95°C for 15 minutes, followed by 35 cycles of 94°C for 30 seconds, 54°C for 30 seconds and a final elongation at 72°C for 10 minutes.

The PCR products were checked on a 1.5% agarose gel and quantified with the Invitrogen Qubit 4 fluorometer (Thermo Fisher Scientific Inc., USA). Afterwards, all the PCR products were pooled in one tube for next-generation sequencing. Sequencing was performed on the Illumina NovaSeq 6000 platform with 2*150 paired-end reads by Genomics Co., Taipei, Taiwan.

**Data processing and analysis**

The Illumina raw reads were demultiplexed by Genomics Co., Taipei, Taiwan. *FastQC* (v.0.11.9; https://github.com/s-andrews/FastQC) was used to check quality. The forward and reverse primers of the demultiplexed reads were trimmed using *Cutadapt* (version 4.2; Martin (2011)). The *USEARCH* platform (v.11.0.667; Edgar (2010)) was used to verify if the primer sequences were completely removed from the demultiplexed reads. The "denoised-paired" function was used to create an amplicon sequence variant (ASV) data output from the demultiplexed reads. The "denoise-paired" function in *QIIME2* (v.2023.2.0; Bolyen et al. (2019)) can automatically trim, filter, denoise, merge reads and remove chimeric reads in one step. The maximum expected error of forward and reverse reads was set to 1.0. The reads with a quality score of less than 20 were truncated. The minimum overlap length for the forward and reverse reads merger was set to 16 bp. Other parameters followed the default settings in the "denoise-paired" function. No reads were trimmed or truncated during the ASV creation process. The ASV output was then further clustered through the "cluster-features-de-novo" function provided by *QIIME 2*. ASVs with more than 97% sequence identity were clustered into one operational taxonomic unit (OTU). The sequences that were shorter than 100 bp in the ASV and OTU results were discarded. Taxonomic assignments were conducted using *Constax* (v.2.0.18; Liber et al. (2021)) against the MIDORI database (v.GB250; Machida et al. (2017)). The R package *phyloseq* was used to analyse the preprocessed sequencing data (v.1.40.0; McMurdie and Holmes (2013)). The piechart figure was produced with *ggplot2* (v.3.4.2; Villanueva and Chen (2019)). Lowest taxon level-annotation of each OTU was extracted to perform a secondary species mapping to the GBIF Backbone Taxonomy (GBIF Secretariat 2011) on 05-12-2023 using the "name_backbone_checklist" function in R package *rgbif* (v.3.7.7; Chamberlain et al. (2022), R Core Team (2023)). The map was visualised using the R packages *ggplot2* (v.3.4.2; Villanueva and Chen (2019)) and annotated using *ggspatial* (v.1.1.9; https://paleolimbot.github.io/ggspatial/), *metR* (v.0.14.0; https://github.com/eliocamp/metR) and *ggrepel* (v.0.9.3; https://CRAN.R-project.org/package=ggrepel).

**Open data and code**

Two datasets were associated with this study: DNA sequence data and occurrence data (see Data resources). We converted the occurrence data into Darwin Core Archive standard (Darwin Core Task Group 2009) and validated the datasheet using the GBIF Data Validator (Global Biodiversity Information Facility 2017). We then published the dataset containing one occurrence core (i.e. foundational part of the dataset with information about each occurrence) and one DNA-derived data extension (Hoh 2023) using the Integrated Publishing Toolkit (IPT) of GBIF installed under the Taiwan Biodiversity Information Facility (TaiBIF). We have included three supplementary files to help describe the dataset. They are the attributes of the sampling event (Suppl. material 1), the water quality measurements (Suppl. material 2) and the relationship of each technical sample to the sampling event (Suppl. material 3). These files were attached as the current GBIF data model schema does not support event core matching with a DNA-derived data extension. All source code used in the project can be found in the project's GitHub repository.

## Geographic coverage

### Description

We selected four sites along the Houdong River (猴洞坑), Jiaoxi Township, Yilan County, Taiwan (Table 1) for water sample collections and in-situ water quality measurements: Upstream waterfall (WF), downstream river (FR), estuary (ES) and river mouth (RM). These four sites spanned a river length of 6.5 km.

### Coordinates

24.824 and 24.871 Latitude; 121.768 and 121.846 Longitude.

## Taxonomic coverage

### Description

**Figure 2.**
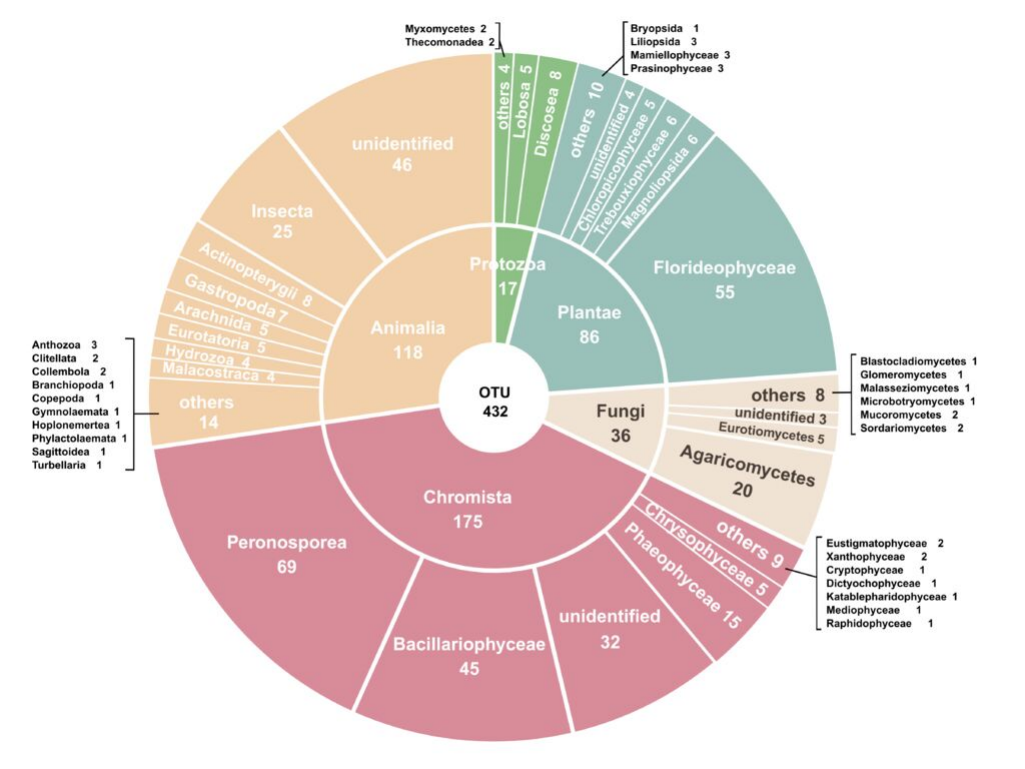
The taxonomic ranking of 432 classified operational taxonomic units (OTUs). The colours represent different kingdoms.

We detected eukaryotic organisms in the water samples using the COI mitochondrial gene. A total of 2,736 OTUs were identified and 421 of the OTUs were assigned to at least the kingdom level using the MIDORI database (v.GB250; Machida et al. (2017); Fig. 2). On GBIF, this dataset (Hoh 2023) consists of a total of 6,297 occurrences with 22% (1,356 occurrences; last accessed 05-12-2023) having a taxon match on the GBIF Backbone Taxonomy, with remaining occurrences being assigned as *incertae sedis* (i.e. taxa unknown). The species occurrence dataset was standardised and presented in GBIF annotated Darwin Core Archive (see Data resources), grouped by sampling events (i.e. sites and identifiable via the *eventID* column).

### Taxa included

**Table:** 

Rank	Scientific Name	
kingdom	Animalia	
kingdom	Chromista	
kingdom	Fungi	
kingdom	Plantae	
kingdom	Protozoa	
phylum	Amoebozoa	
phylum	Bryophyta	
phylum	Cryptophyta	
phylum	Nemertea	
phylum	Sulcozoa	
phylum	Annelida	
phylum	Bryozoa	
phylum	Gastrotricha	
phylum	Ochrophyta	
phylum	Tracheophyta	
phylum	Arthropoda	
phylum	Chaetognatha	
phylum	Glomeromycota	
phylum	Oomycota	
phylum	Zygomycota	
phylum	Ascomycota	
phylum	Chlorophyta	
phylum	Haptophyta	
phylum	Platyhelminthes	
phylum	Basidiomycota	
phylum	Chordata	
phylum	Mollusca	
phylum	Rhodophyta	
phylum	Blastocladiomycota	
phylum	Cnidaria	
phylum	Mycetozoa	
phylum	Rotifera	

## Temporal coverage

### Notes

This was a one-time sampling of water samples and corresponding water quality parameters on 28-04-2022.

## Usage licence

### Usage licence

Other

### IP rights notes

Datasets produced by the current work are licensed under a Creative Commons Attribution (CC-BY) 4.0 Licence.

## Data resources

### Data package title

eDNA along Houdong riverine zonation in Taiwan

### Number of data sets

2

### Data set 1.

#### Data set name

eDNA along riverine zonation of Houdong River, Yilan, Taiwan [Project ID: PRJEB60905]

#### Data format

Genomic Standard Consortium MIxS water package

#### Download URL

https://www.ebi.ac.uk/ena/browser/view/PRJEB60905

#### Data format version

mixs6.1.0

#### Description

DNA sequence data have been deposited on ENA at EMBL-EBI under accession number PRJEB60905 following the Genomic Standard Consortium MIxS standard (Yilmaz et al. 2011). Below are described the nine default columns under the 'Read Files' section on the project (or dataset) page, which can also be obtained from downloading the TSV report on the Project page.

**Data set 1.** 

Column label	Column description
study_accession	The project accession number created by ENA for this submission (PRJEB60905 for this dataset).
sample_accession	The sample accession number created by ENA for this submission. A total of 12 Biosamples (comprised of three replicates from each of the four sampling sites) were registered. Each accession from the link https://www.ebi.ac.uk/ena/browser/view/[sample_accession] describes basic information about the sample following the MIxS standard.
experiment_accession	The experiment accession number created by ENA for this submission. A total of 12 Experiments were registered. Each accession from the link https://www.ebi.ac.uk/ena/browser/view/[experiment_accession] describes sequencing instrument and library-associated information.
run_accession	The run accession number created by ENA for this submission. A total of 12 Runs were registered. Each accession from the link https://www.ebi.ac.uk/ena/browser/view/[run_accession] contains read and base count information.
tax_id	Taxon ID in ENA. Since this is a metabarcoding study, all entries are 256318, which corresponds to 'metagenome'.
scientific_name	Since this is a metabarcoding study, scientific name is not applicable and hence all entries are 'metagenome'.
fastq_ftp	The FTP link to download DNA reads obtained from each Run. This is the ENA Archived Generated File as described here. The format of the file is a gunzip-compressed FASTQ file. Two FTP links were provided that separate the forward and reverse reads from each paired experiment by [run_accession]_1.fastq.gz and [run_accession]_2.fastq.gz, respectively.
submitted_ftp	The FTP link to download DNA reads uploaded to ENA by the submitter before automation curation resulting to fastq_ftp.
bam_ftp	The FTP link to download the BAM file of each Run.

### Data set 2.

#### Data set name

eDNA along Houdong riverine zonation in Taiwan

#### Data format

Darwin Core Archive

#### Download URL

https://www.gbif.org/dataset/2615342d-7349-4e75-ae34-cda6cb403e2e ; https://ipt.taibif.tw/archive.do?r=houdongkeng_water_edna

#### Data format version

2021-07-15

#### Description

There are two links in the Download URL. The first links to the download page of the GBIF annotated Darwin Core Archive and the second links to the Source Darwin Core Archive from the TaiBIF IPT. The second link is provided because the DNA-derived data extension associated with the occurrence datasheet is not available through the GBIF-annotated Darwin Core Archive download option, although the extension is included in the source archive available either through the GBIF webpage for the dataset or directly from the TaiBIF IPT. Downloading from both links gives GZ-compressed files containing the occurrence core and DNA-derived data extension files in TXT format. The below table describes a total of 76 data fields from both the occurrence core and DNA derived data extension, sorted alphabetically. The data field descriptions are written as listed in the List of Darwin Core terms (accessed April 2023; Darwin Core Task Group (2009)), but modified as needed if applicable to the current study context. The occurrence core datasheet can also be downloaded via GBIF API-based tools such as *rgbif* (Chamberlain et al. 2022) for further analyses.

**Data set 2.** 

Column label	Column description
ampliconSize	The length of the amplicon in basepairs.
amplificationReactionVolume	PCR reaction volume
amplificationReactionVolumeUnit	Unit used for PCR reaction volume.
basisOfRecord	The specific nature of the data record.
class	The full scientific name of the class in which the taxon is classified.
concentration	Concentration of DNA (weight ng/volume µl).
concentrationUnit	Unit used for concentration measurement.
continent	The name of the continent in which the Location occurs.
coordinateUncertaintyInMetres	The horizontal distance (in metres) from the given decimalLatitude and decimalLongitude describing the smallest circle containing the whole of the Location.
country	The name of the country in which the Location occurs.
countryCode	The standard code for the country in which the Location occurs.
county	The full, unabbreviated name of the smaller administrative region in which the Location occurs.
datasetName	The name identifying the dataset from which the record was derived.
dateIdentified	The date on which the subject was determined as representing the Taxon.
day	The integer day of the month on which the Event occurred.
decimalLatitude	The geographic latitude (in decimal degrees, using the spatial reference system given in geodeticDatum) of the geographic centre of a Location.
decimalLongitude	The geographic longitude (in decimal degrees, using the spatial reference system given in geodeticDatum) of the geographic centre of a Location.
DNA_sequence	The DNA sequence.
env_broad_scale	The major environmental system from which the sample came. ENVO's biome subclasses determined in https://ontobee.org/ontology/ENVO.
env_local_scale	The entity in which the sample's local vicinity, smaller spatial grain than the entry in env_broad_scale. ENVO's biome subclasses determined in https://ontobee.org/ontology/ENVO.
env_medium	Environmental material immediately surrounded the sample prior to sampling, using subclasses of ENVO’s environmental material class determined in https://ontobee.org/ontology/ENVO.
eventDate	The date-time when the event was recorded (i.e. water sampling time).
eventID	An identifier associated with an sampling event.
eventTime	The time when the event was recorded (i.e. water sampling time).
experimental_factor	The variable aspects of an experiment design to describe the experiment. The ontology terms determined from Experimental Factor Ontology (EFO).
family	The full scientific name of the family in which the taxon is classified.
genus	The full scientific name of the genus in which the taxon is classified.
geodeticDatum	The full scientific name of the genus in which the taxon is classified.
habitat	A category or description of the habitat in which the Event occurred. Using subclasses of ENVO’s environmental material class determined in https://ontobee.org/ontology/ENVO.
higherClassification	Taxa names terminating at the rank immediately superior to the taxon referenced in the taxon record. Current metabarcoding study was targeting the eukaryotic organism and hence the entry is "Eukaryota".
identificationReferences	Publication reference used in the Identification.
identificationRemarks	Comments or notes about the Identification.
kingdom	The full scientific name of the kingdom in which the taxon is classified.
lib_layout	Specify whether to expect single, paired or other configuration of reads.
licence	A legal document giving official permission to do something with the resource.
locationID	An identifier for the set of location information as listed in Table 1.
materialSampleID	An identifier for the MaterialSample.
methodDeterminationConcentrationAndRatios	Method used for concentration measurement.
month	The integer month in which the Event occurred.
nucl_acid_amp	A link to electronic resource that describes the PCR amplification of specific nucleic acids.
nucl_acid_ext	A link to electronic resource that describes the material separation to recover the nucleic acid fraction from a sample.
occurrenceID	An identifier for the Occurrence. Format: [samp_name]:OTU[number].
occurrenceStatus	A statement about the presence or absence of a Taxon at a Location.
order	The full scientific name of the order in which the taxon is classified.
organismQuantity	Number of reads of this OTU in the sample.
organismQuantityType	DNA sequence reads.
otu_class_appr	Cutoffs and approach used when clustering the "species-level" OTUs.
otu_db	Reference database for "species-level" OTUs.
otu_seq_comp_appr	Tool and thresholds used to compare sequences when computing "species-level" OTUs.
pcr_cond	Description of reaction conditions and components of PCR.
pcr_primer_forward	Forward PCR primer that were used to amplify the sequence of the targeted gene.
pcr_primer_name_forward	Name of the forward PCR primer used.
pcr_primer_name_reverse	Name of the reverse PCR primer used.
pcr_primer_reference	Reference for the PCR primers that were used to amplify the sequence of the targeted gene.
pcr_primer_reverse	Reverse PCR primer that were used to amplify the sequence of the targeted gene.
pcr_primers	PCR primers that were used to amplify the sequence of the targeted gene, locus or subfragment.
phylum	The full scientific name of the phylum or division in which the taxon is classified.
preparations	Preparations methods for the sample.
project_name	Name of the project within which the sequencing was organised.
rightsHolder	The organisation owning or managing rights over the resource.
samp_mat_process	The processing applied to the sample after retrieving the sample from environment.
samp_name	Unique sample name for each sample. Starts with abbreviation of sampling site as listed in Table 1 and ends with sample replicate number.
samp_size	Amount of sample (volume) that was collected.
sampleSizeUnit	DNA sequence reads.
sampleSizeValue	Total number of reads in the sample.
samplingProtocol	The names of, references to, or descriptions of the methods or protocols used during an Event.
scientificName	The full scientific name in the lowest level taxonomic rank that can be determined. The content in this field was obtained from secondary mapping of the lowest taxonomic rank in verbatimIdentification to the GBIF Backbone Taxonomy (see Methodology).
seq_meth	Sequencing method used.
size_frac	Filtering pore size used in sample preparation.
target_gene	Targeted gene or locus name for marker gene studies.
tax_ident	The phylogenetic marker(s) used to assign an organism name.
taxonRank	The taxonomic rank of the most specific name in the scientificName.
type	The nature or genre of the resource.
verbatimIdentification	The taxonomic identification of otu_db.
verbatimLocality	The original textual description of the place.
year	The four-digit year in which the Event occurred, according to the Common Era Calendar.

## Supplementary Material

25D8B6FC-8BC0-5538-B7C9-AD97AA8E7BAB 10.3897/BDJ.12.e116921.suppl1 Supplementary material 1 Sampling event data Data type event Brief description A TSV datasheet in Darwin Core Archive format describing the four sampling events along the river. File: oo_947548.tsv https://binary.pensoft.net/file/947548 Daphne Z. Hoh

4EFB0C43-96A4-5B9B-9429-0F7B4C105D85 10.3897/BDJ.12.e116921.suppl2 Supplementary material 2 Water quality measurements in each sampling event Data type measurement or fact Brief description A TSV datasheet in Darwin Core Archive format describing the water quality measurements in the four sampling events along the river. File: oo_947549.tsv https://binary.pensoft.net/file/947549 Daphne Z. Hoh

818C017F-5F57-5F36-BCEE-0E85431F1298 10.3897/BDJ.12.e116921.suppl3 Supplementary material 3 Sample relationship Data type resource relationship Brief description A TSV datasheet in Darwin Core Archive format describing the relationship of each technical sample to the four sampling events. File: oo_947550.tsv https://binary.pensoft.net/file/947550 Daphne Z. Hoh

## Figures and Tables

**Table 1.** Coordinates of the four sampling sites.

Station	North	East
猴洞坑瀑布 | Upstream waterfall (WF)	24.843580	121.781830
猴洞溪 | Downstream river (FR)	24.835094	121.799932
下埔排水線 | Estuary (ES)	24.835900	121.818660
竹安出海口 | River mouth (RM)	24.840520	121.826640
